# Novel *MSH2* frameshift variant (c.579delG) in a patient with suspected Lynch syndrome in China

**DOI:** 10.3389/fmed.2026.1714599

**Published:** 2026-03-30

**Authors:** Haichun Ni, Xiufang Wang, Yi Zhu, Chengzhi He, Hengfei Li, Aiping Deng, Fei You, Juyi Li, Yi Hu

**Affiliations:** 1 Department of Pathology, The Central Hospital of Wuhan, Tongji Medical College, Huazhong University of Science and Technology, Wuhan, Hubei, China; 2 Department of Pain, The Central Hospital of Wuhan, Tongji Medical College, Huazhong University of Science and Technology, Wuhan, Hubei, China; 3 Department of Anesthesiology, The Central Hospital of Wuhan, Tongji Medical College, Huazhong University of Science and Technology, Wuhan, China; 4 Department of Gastrointestinal Surgery, The Central Hospital of Wuhan, Tongji Medical College, Huazhong University of Science and Technology, Wuhan, China; 5 Department of Infectious Diseases, Hubei Provincial Hospital of Traditional Chinese Medicine, Wuhan, Hubei, China; 6 Department of Pharmacy, The Central Hospital of Wuhan, Tongji Medical College, Huazhong University of Science and Technology, Wuhan, Hubei, China; 7 Department of Rehabilitation, The Central Hospital of Wuhan, Tongji Medical College, Huazhong University of Science and Technology, Wuhan, Hubei, China; 8 Department of Pulmonary and Critical Care Medicine, The Central Hospital of Wuhan, Tongji Medical College, Huazhong University of Science and Technology, Wuhan, Hubei, China

**Keywords:** colorectal cancer, Lynch syndrome, *MSH2*, *PMS2*, whole-exome sequencing

## Abstract

**Purpose:**

To identify genetic variants in Chinese families with colorectal cancer.

**Methods:**

Expression of mismatch-repair proteins was assessed via immunohistochemistry in three probands. Genetic variants were identified using whole-exome sequencing. *In silico* analyses were performed to assess the pathogenicity of these variants and their impacts on protein three-dimensional (3D) structure.

**Results:**

Two known missense variants (*PMS2*:NM_000535:exon11:c.1847T>C:p.V616A and *PMS2*:NM_000535:exon14:c.2444C>T:p.S815L) and a novel frameshift variant (*MSH2*:NM_000251:exon3:c.579delG:p.Q193fs) were found in families 1, 2, and 3, respectively. Protein 3D modeling showed that the c.2444C>T variant in the PMS2 protein locally altered the protein structure. In the wild-type PMS2 protein, S815 formed hydrogen bonds with N708 (3.1 Å), A811 (2.9 Å and 3.2 Å), and C812 (3.6 Å), whereas the mutant L815 did not form hydrogen bonds with nearby residues. The c.579delG variation in the MSH2 protein led to truncation from 934 amino acids to 212 amino acids, altered the sequence of the Domain 2 region, and caused the loss of Domains 3, 4, and 5.

**Conclusion:**

The two missense variants of the *PMS2* gene (*PMS2*:NM_000535:exon11:c.1847T>C:p.V616A and *PMS2*:NM_000535:exon14:c.2444C>T:p.S815L) were considered variants of uncertain significance, whereas the novel frameshift variant of the *MSH2* gene (*MSH2*:NM_000251:exon3:c.579delG:p.Q193fs) was considered likely pathogenic.

## Introduction

Lynch syndrome (LS) is an autosomal dominant genetic disorder caused by pathogenic mutations in DNA mismatch repair (MMR) genes: *MLH1*, *MSH2*, *MSH6*, and *PMS2*, accounting for approximately 2–5% of all colorectal cancer (CRC) cases (1), with CRC representing the second leading cause of cancer-related deaths (2). Loss of MMR protein expression indicates deficient MMR activity (dMMR), resulting in the accumulation of DNA-replication errors and genomic instability, thereby promoting carcinogenesis (3). *MSH2* and *MLH1* mutations are the most common, accounting for 40 and 50% of cases, respectively (4), whereas approximately 10–20% of mutations in MMR genes involve *PMS2* and *MSH6* (5). Individuals carrying mutations in MMR genes are predisposed to various cancers, including colorectal, gastric, small intestine, biliary tract, endometrial, ovarian, renal pelvis, bladder, ureteral, skin, and brain cancers (6).

In recent years, the emergence of next-generation sequencing and the continual advancement of analytical techniques have led to the discovery of numerous variants affecting the functions of MMR proteins. The estimated prevalence of LS in the general population is 1:440. Because individuals with this syndrome are at risk of developing various cancers beyond CRC, identifying pathogenic variants and implementing appropriate monitoring are crucial for effective health management (7, 8).

Identifying carriers of mutations in MMR genes is essential for optimizing cancer monitoring and prevention. Several clinical trials have demonstrated the benefits of anti-PD1 therapy in CRC patients with microsatellite instability-high (MSI-H)/dMMR, showing overall response rates of 40–60% (9). The National Comprehensive Cancer Network guidelines recommend universal MMR or MSI testing for all CRC patients, and advise that MSI-H/dMMR patients receive pembrolizumab or nivolumab, alone or in combination with ipilimumab, as first-line treatment (4). Consequently, accurate diagnosis of LS is critical for determining precise treatment of patients and providing genetic counseling to their family members.

This study aimed to identify the potential genetic factors underlying the CRC development in three unrelated Chinese families clinically diagnosed with suspected LS. We detected two known missense variants in the *PMS2* gene (*PMS2*:NM_000535:exon11:c.T1847C:p.V616A and *PMS2*:NM_000535:exon14:c.C2444T:p.S815L) and a novel frameshift variant in the *MSH2* gene (*MSH2*:NM_000251:exon3:c.579delG:p.Q193fs). We evaluated the impacts of these variants on protein structure and function via bioinformatics approaches and provided recommendations to guide chemotherapy selection for the treatment of late-stage LS patients.

## Methods and materials

### Patients

Three CRC patients who underwent partial colectomy were included in this study. Written informed consent was obtained from all the participants. Medical records were collected from our clinics. The study was approved by the Ethics Committee of the Central Hospital of Wuhan.

### Immunohistochemistry

Colorectal pathological samples were processed for standard histopathological analysis via hematoxylin–eosin (H&E) staining. To assess dMMR, immunohistochemistry (IHC) was performed for MLH1, PMS2, MSH2, and MSH6 by using the corresponding mouse monoclonal antibodies (1, 10).

### Next-generation sequencing

Total DNA was isolated from formalin-fixed paraffin-embedded samples by using Qiagen DNeasy Kits. Whole-exome sequencing was performed on the Illumina HiSeq-2500 platform. Sequence reads were aligned and analyzed using hg19 as the reference genome (11, 12). Genomic variants were assessed using the Genome Aggregation Database and ClinVar, and their pathogenicity was classified according to the standards of the American College of Medical Genetics and Genomics.

### Sanger sequencing

Three sets of PCR primers were used for the detection of variants. Forward and reverse primers were 5′-TTAGTTCATCTTCGGCTGCTTG-3′ and 5′-GGACAGGGGCTCGCAGGAACAT-3′, respectively, for Family 1; 5′-ACACAGATGCTCAGCTACGA-3′ and 5′-CGGCCTCAGATGTTCATCCT-3′ for Family 2; and 5′-CCATTGGTGTTGTGGGTGTT-3′ and 5′-AAGAGCCTTTCCTAGGCCTG-3′ for Family 3.

### Three-dimensional modeling of protein structures

The amino acid sequences of the MSH2 and PMS2 proteins were obtained from GenBank. Three-dimensional (3D) structures of MSH2 and PMS2 were modeled using I-TASSER^1^ and visualized using PyMOL.^2^

## Results

### Clinical phenotypes

**Figure 1:**
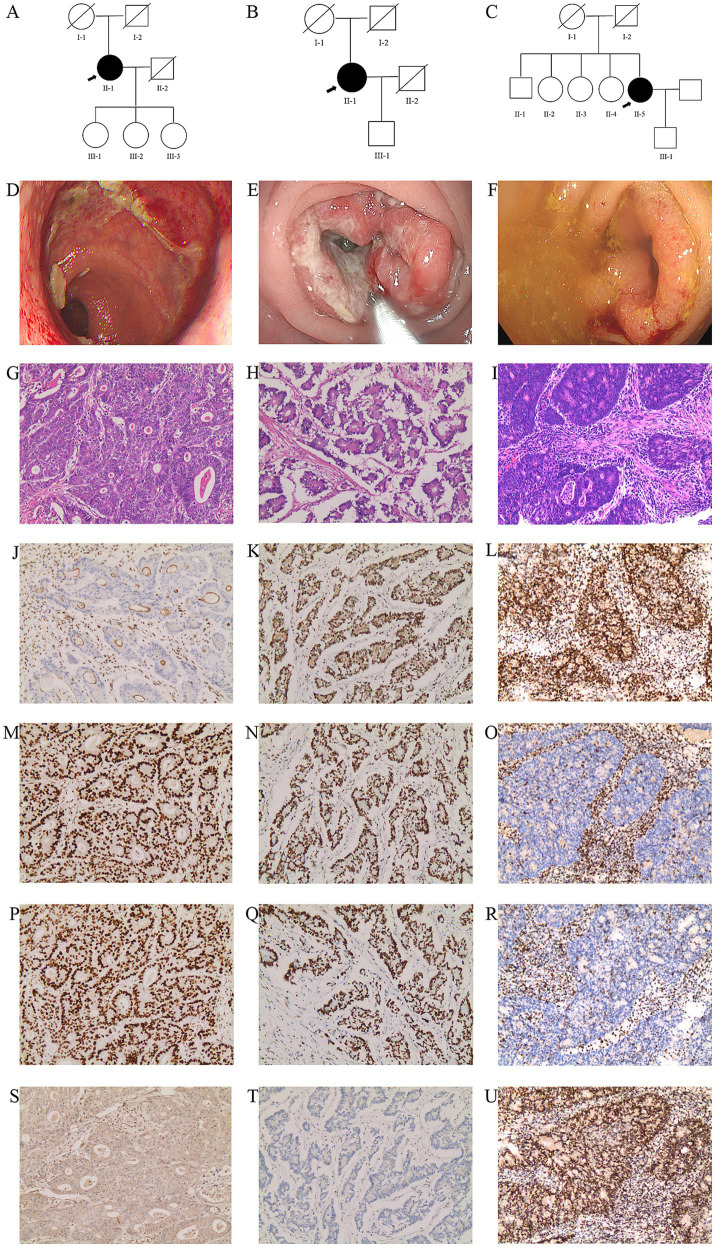
Three families with suspected LS. **(A–C)** Pedigrees of the three probands in Family 1 **(A)**, Family 2 **(B)**, and Family 3 **(C)**. Arrows indicate the probands. Squares and circles denote males and females, respectively. Filled and empty symbols represent patients with suspected LS and unaffected individuals, respectively, and crosses indicate deceased individuals. **(D)** Follow-up colonoscopy image of the proband in Family 1 after surgery for distal ileocecal intussusception. **(E)** Large tumor with surface ulceration in the right colon of the proband in Family 2. **(F)** Sigmoid-colon tumor with surface congestion and ulceration, partially obstructing the lumen, in the proband in Family 3. **(G–I)** Histological analyses of the cancer tissues in Family 1 **(G)**, Family 2 **(H)**, and Family 3 **(I)**. **(J–U)** Immunohistochemical staining for MLH1 **(J–L)**, MSH2 **(M–O)**, MSH6 **(P–R)**, and PMS2 **(S–U)**; images from left to right correspond to Families 1 **(J,M,P,S)**, 2 **(K,N,Q,T)**, and 3 **(L,O,R,U)**, respectively.

The proband in Family 1 (II-1, 72 years old; Figure 1A) was diagnosed with colon cancer following abdominal discomfort, dull pain, and changes in stool consistency. He underwent a full abdominal computed tomography scan and pathological examination. The causes of death of both parents are unknown.

The proband in Family 2 (II-1, 75 years old; Figure 1B) underwent colonoscopy and pathological examination due to recurrent abdominal distension and melena, and was diagnosed with colon cancer. The causes of death of both parents are unknown.

The proband in Family 3 (II-5, 66 years old; Figure 1C) underwent colonoscopy and pathological examination due to abdominal distension and pain, and was diagnosed with sigmoid colon cancer. At the age of 54, she was diagnosed with endometrial cancer. The causes of death of both parents are unknown.

Figure 1D shows the colonoscopy image of the proband in Family 1 after removal of an intestinal lesion. Figures 1E,F show the large tumors in the intestines of the probands in families 2 and 3, respectively.

### Histopathological analysis

In Family 1, H&E staining revealed poorly differentiated adenocarcinoma of the colon with the cancer infiltrating the subserosal layer (Figure 1G). In Family 2, a moderately differentiated adenocarcinoma of the right colon was observed, with the cancer penetrating the muscularis propria and infiltrating the subserosal fibrofatty tissue (Figure 1H). In Family 3, H&E staining showed poorly differentiated adenocarcinoma of the descending colon with invasion into the subserosal layer (Figure 1I).

IHC staining of tumor cells showed loss of nuclear expression of MLH1 (Figure 1J) and PMS2 (Figure 1S), MSH2 (Figure 1M) and MSH6 (Figure 1P) was retained in Family 1. In Family 2, staining revealed loss of nuclear expression of PMS2 (Figure 1T), whereas the nuclear expression of MLH1 (Figure 1K), MSH2 (Figure 1N), and MSH6 (Figure 1Q) was retained. In Family 3, loss of nuclear expression was observed for MSH2 (Figure 1O) and MSH6 (Figure 1R), whereas the nuclear expression of MLH1 (Figure 1L) and PMS2 (Figure 1U) was preserved.

### Whole-exome and Sanger sequencing

Details of the whole-exome sequencing for the three probands are shown in Table 1. In Family 1, a known missense variant, *PMS2*:NM_000535:exon11:c.1847T>C:p.V616A (rs1583314800), was identified. This variant is rare, with a very low allele frequency (*G* = 0.000002, 1/595,684, GnomAD_exomes; *G* = 0.00000, 0/10,680, ALFA). In ClinVar, this variant is classified as a variant of uncertain significance (VUS); however, multiple bioinformatics prediction tools indicated that it is likely deleterious: MutationTaster score = 1 (deleterious), PolyPhen-2_HVAR score = 0.863 (possibly deleterious), and PolyPhen-2_HDIV score = 0.992 (deleterious).

**Table 1:** The details of whole-exome sequencing of the three probands.

Sample	Proband 1	Proband 2	Proband 3
Total	51,647,962 (100%)	81,721,846 (100%)	72,058,206 (100%)
Mapped	51,580,332 (99.87%)	81,673,692 (99.94%)	72,026,420 (99.96%)
Initial_bases_on_target	60,456,963	60,456,963	60,456,963
Total_effective_yield (Mb)	7600.57	12206.18	10760.92
Effective_yield_on_target (Mb)	5211.59	8287.62	7281.91
Average_sequencing_depth_on_target	86.20	137.08	120.45
Coverage_of_target_region	99.3%	99.5%	99.4%
Fraction_of_target_covered_with_at_least_100×	33.1%	57.6%	51.4%
Fraction_of_target_covered_with_at_least_50×	66.1%	82.3%	79.7%
Fraction_of_target_covered_with_at_least_4×	98.3%	99.0%	98.9%

In Family 2, a known missense variant, *PMS2*:NM_000535:exon14:c.2444C>T:p.S815L (rs587779338), was identified. This variant is rare, with a very low allele frequency (*A* = 0.00003, 1/29,636, ExAC; *A* = 0.00000, 0/14,050, ALFA). In ClinVar, this variant is classified as likely pathogenic, pathogenic, or uncertain significance. Furthermore, multiple bioinformatics prediction tools indicated that it is deleterious: MutationTaster score = 1, PolyPhen-2_HVAR score = 0.999, PolyPhen-2_HDIV score = 1.0, SIFT score = 0.0, and LRT score = 0.000000.

**Figure 2:**
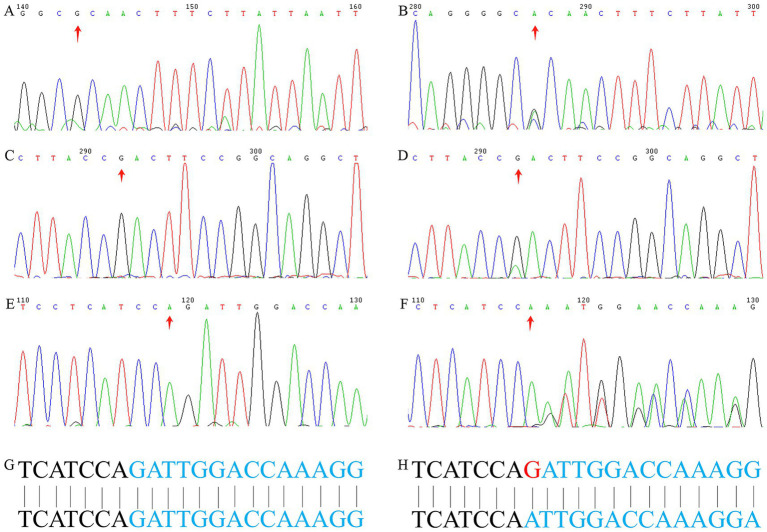
Confirmation of the variants via Sanger sequencing using blood and tumor tissue DNA. **(A)** Wild-type and **(B)** mutant alleles of the *PMS2* gene (c.1847T>C) in Family 1, respectively. **(C)** Wild-type and **(D)** mutant alleles of the *PMS2* gene (c.2444C>T) in Family 2, respectively. **(E)** Wild-type and **(F)** mutant alleles of the *MSH2* gene (c.579delG) in Family 3, respectively. **(G)** Wild type base sequence, **(H)** mutant type base sequence.

A novel frameshift variant, *MSH2*:NM_000251:exon3:c.579delG:p.Q193fs, was identified in Family 3 and has not been reported in any database. All the three variants were validated via Sanger sequencing using peripheral blood DNA (Figure 2).

The two missense variants (*PMS2*:NM_000535:exon11:c.1847T>C:p.V616A and *PMS2*:NM_000535:exon14:c.2444C>T:p.S815L) were classified as VUS (PP3 + PP4), whereas the novel frameshift deletion variant (*MSH2*:NM_000251:exon3:c.579delG:p.Q193fs) was classified as pathogenic (PVS1 + PM2 + PM6 + PP4).

### Modeling of protein structures

**Figure 3:**
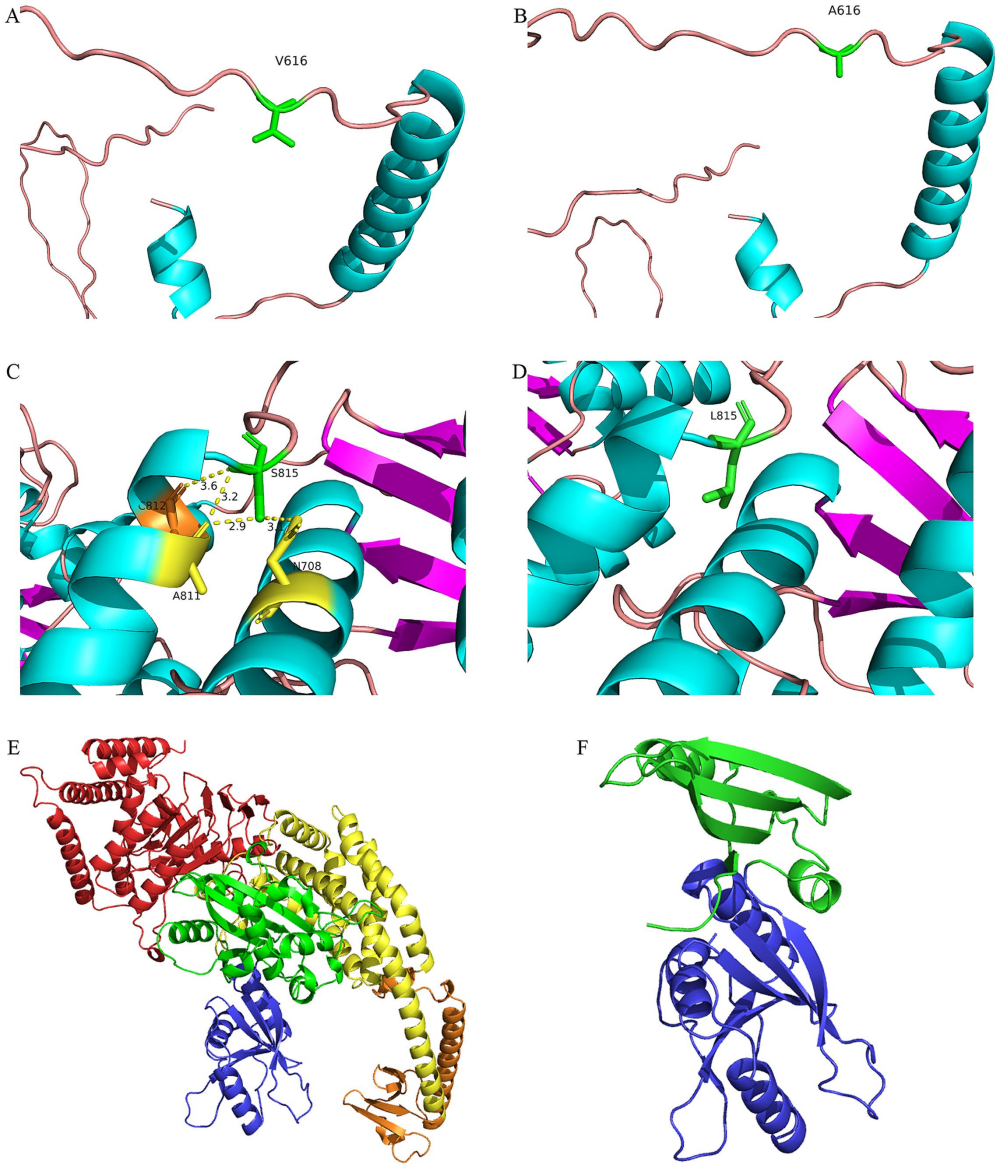
Modeling of protein structures. Local structures of **(A)** wild-type (V616) and **(B)** mutant (A616) PMS2 proteins, respectively. Local structures of **(C)** wild-type (S815) and **(D)** mutant (L815) PMS2 proteins, respectively. Overall structures of **(E)** wild-type and **(F)** mutant (c.579delG) MSH2 proteins, respectively. Domains 1–5 are colored blue, green, yellow, orange, and red, respectively. Structure modeling was performed using I-TASSER.

The c.1847T>C variant in *PMS2* resulted in the substitution of valine (Figure 3A) at position 616 with alanine (Figure 3B). This variant is located in the loop region of PMS2 and did not have a significant impact on the predicted overall or local protein structure. The c.2444C>T variant caused the replacement of serine (Figure 3C) at position 815 with leucine (Figure 3D), changing a polar amino acid to a non-polar one. The 3D protein model indicated that this variant locally affected the PMS2 structure: the wild-type S815 formed hydrogen bonds with N708 at 3.1 Å, with A811 at 2.9 Å and 3.2 Å, and with C812 at 3.6 Å, whereas the mutant L815 did not form hydrogen bonds with surrounding amino acids.

The c.579delG variant resulted in a truncated MSH2 protein, reducing its length from 934 to 212 amino acids, altering the sequence of the Domain 2 region, and causing the loss of Domains 3, 4, and 5 (Figures 3E, F).

## Discussion

LS is considered an adult-onset genetic disorder, with the average age of CRC onset ranging from 44 to 66 years. The risk of developing CRC in LS patients before the age of 70 is 52–82%, which is 9.5–15 times higher than that in the general population. The majority of LS-associated mutations are inherited from affected parents, who may or may not exhibit clinical symptoms (6, 13). Traditional diagnostic criteria for LS, such as the Amsterdam II criteria and the revised Bethesda guidelines, rely on family history of cancer; however, such information is not always available or reliable. In China, the one-child policy over the past few decades has further reduced the size of family genealogies, limiting their availability. Consequently, combining family history with genetic testing provides a more reliable approach for diagnosing LS (14, 15).

Universal genetic screening should be conducted for all newly diagnosed cancer patients to assess genetic etiology in both adults and children. With recent technological advancements, multi-gene panel testing of tumor DNA may replace microsatellite instability and IHC as first-line detection methods. Genetic testing in early-onset CRC enables optimal medical management for patients and their families, including avoiding unnecessary screening, alleviating anxiety in unaffected relatives, and reducing CRC mortality by over 60% (15–17).

Genetic testing for hereditary cancers often fails to identify clearly pathogenic variants, and misclassification of genetic variants can significantly impact high-risk families by leading to inappropriate clinical recommendations. Therefore, accurate identification of pathogenic variants within families is crucial for proper individual risk assessment. A large proportion of genetic variants in patients with suspected LS are classified as VUS, with *MLH1*, *MSH2*, and *MSH6* variants accounting for 32, 18, and 38% of such cases, respectively (18). Most alterations in *MSH2*, *MLH1*, and *MSH6* are truncating variants, primarily nonsense or frameshift mutations. Consequently, IHC detection of MMR protein expression serves as a rapid and cost-effective screening tool for MSI and LS (19). Most VUSs are missense mutations or small in-frame deletions. A substantial number of rare missense mutations exist in the human gene pool, approximately 70% of which are at least mildly deleterious (20). Overall, the proportion of VUSs in LS ranges from one-fifth to one-third of all detected unique variants (18).

*PMS2* variants account for 6–15% of LS cases, and individuals with heterozygous variants have an estimated 11–20% risk of developing CRC or endometrial cancer by the age of 70 (21). In the present study, our findings suggested that the two missense variants (*PMS2*:NM_000535:exon11:c.1847T>C:p.V616A and *PMS2*:NM_000535:exon14:c.2444C>T:p.S815L) are VUSs; however, multiple bioinformatics prediction tools indicated that both sites are likely deleterious. In particular, the *PMS2*:c.2444C>T variant disrupts hydrogen bonding between L815 and surrounding amino acids, potentially affecting PMS2 protein stability and reducing its binding affinity to MLH1. Suerink et al. (22) have reported this variant in a family with CRC and classified it as VUS, whereas van der Klift et al. (21) have classified it as pathogenic based on *in vitro* evidence of dMMR. Similarly, González-Acosta et al. (23) have found that *PMS2*:c.2444C>T decreases MLH1/PMS2 protein levels and impairs MMR activity in functional assays, also supporting its pathogenic classification. Anyway, Family 1 and 2 may also be defined as Lynch like syndrome. Therefore, further in-depth research is needed to clarify the relationship between these two variants and LS, and large-cohort studies are needed to confirm their pathogenicity.

Recent studies suggest that the prevalence of LS in the general population is higher than previously estimated. Analysis of colon-cancer family-registry data indicates that the carrier frequency of pathogenic germline mutations in any of the four MMR genes is approximately 1/279, with pathogenic *MSH2* mutations occurring at an estimated frequency of 1/758 (6). In the present study, a novel *MSH2* variant (*MSH2*:NM_000251:exon3:c.579delG:p.Q193fs) was identified and classified as pathogenic, due to the lack of functional experiments, we defined it as likely pathogenic. MSH2 functions in repairing DNA-replication errors, and the frameshift variant results in the loss of Domains 3, 4, and 5. Dysfunction of MSH2 leads to the accumulation of mutations, genomic instability, and ultimately, cancer (24).

In addition to LS, MMR testing has a recognized prognostic and predictive role in sporadic CRC. CRC patients with dMMR generally have a better prognosis but rarely benefit from fluorouracil-based chemotherapy (25). dMMR has also become an important predictor of response to PD-1/PD-L1 inhibitors, highlighting the role of MMR IHC in guiding immunotherapy (26, 27). Clinical trials have shown that CRC patients with MSI-H/dMMR can benefit from anti-PD1 therapy, with overall response rates of 40–60%. Anti-PD1 monoclonal antibodies have been approved as first-line treatment for advanced MSI-H CRC and show durable and stable anti-tumor efficacy in metastatic MSI-H CRC (9, 28). Therefore, patients in this study should prioritize PD-1/PD-L1 inhibitor therapy and avoid fluorouracil-based regimens in later treatment stages.

In conclusion, the two missense variants (*PMS2*:NM_000535:exon11:c.1847T>C:p.V616A and *PMS2*:NM_000535:exon14:c.2444C>T:p.S815L) were classified as VUS, whereas the novel frameshift deletion variant (*MSH2*:NM_000251:exon3:c.579delG:p.Q193fs) was classified as likely pathogenic. Further studies are needed to investigate the mechanisms whereby these three variants contribute to LS.

## Data Availability

The original contributions presented in the study are publicly available. This data can be found here: NCBI SRA, PRJNA1440053.

## Footnotes

1
https://seq2fun.dcmb.med.umich.edu//I-TASSER/

2
https://pymol.org/2/

## References

[1] Yamaguchi M Akabane S Niitsu H Nakahara H Toshida A Mochizuki T . The usefulness of comprehensive genome profiling test in screening of Lynch syndrome independent of the conventional clinical screening or microsatellite instability tests. J Hum Genet. (2025) 70:385–93. doi: 10.1038/s10038-025-01345-x, 40335734 PMC12289520

[2] Wang T Tao Y Gan G Chen L Xu Y Sun F . Application of high-dose-rate endorectal brachytherapy in the treatment of locally advanced rectal cancer. Precis Radiat Oncol. (2025) 9:133–42. doi: 10.1002/pro6.70004, 41164427 PMC12559926

[3] Brignola C Volorio S De Vecchi G Zaffaroni D Dall’Olio V Mariette F . *De novo* germline pathogenic variant in Lynch syndrome: a rare event or the tip of the iceberg? Tumori. (2024) 110:69–73. doi: 10.1177/03008916231197113, 37691472 PMC10851626

[4] Xu Y Li Q Zhao J Ni X Li P Hu W. Case report: complete response to pembrolizumab in a liver metastatic colon adenocarcinoma patient with a novel likely pathogenic germline MSH2 mutation. Front Immunol. (2022) 13:1064488. doi: 10.3389/fimmu.2022.1064488, 36518767 PMC9742472

[5] Liccardo R De Rosa M Rossi GB Carlomagno N Izzo P Duraturo F. Incomplete segregation of MSH6 frameshift variants with phenotype of Lynch syndrome. Int J Mol Sci. (2017) 18:999. doi: 10.3390/ijms18050999, 28481244 PMC5454912

[6] Zajo K Colace SI Mouhlas D Erdman SH. Lynch syndrome-associated colorectal cancer in a 16-year-old girl due to a de novo MSH2 mutation. BMJ Case Rep. (2020) 13:e233935. doi: 10.1136/bcr-2019-233935, 32611652 PMC7332177

[7] Yamamoto G Miyabe I Tanaka K Kakuta M Watanabe M Kawakami S . SVA retrotransposon insertion in exon of MMR genes results in aberrant RNA splicing and causes Lynch syndrome. Eur J Hum Genet. (2021) 29:680–6. doi: 10.1038/s41431-020-00779-5, 33293698 PMC8115629

[8] Dominguez-Valentin M Sampson JR Seppala TT ten Broeke SW Plazzer J-P Nakken S . Cancer risks by gene, age, and gender in 6350 carriers of pathogenic mismatch repair variants: findings from the prospective Lynch syndrome database. Genet Med. (2020) 22:15–25. doi: 10.1038/s41436-019-0596-9, 31337882 PMC7371626

[9] Zhang Q Hu J Zhang Y Li L Wang T Qian X. Case report: a colorectal cancer patient with microsatellite instability-high and MSH2 germline mutation failed to respond to anti-PD-1 immunotherapy. Front Immunol. (2022) 13:953421. doi: 10.3389/fimmu.2022.953421, 35990637 PMC9389357

[10] Appah EO Ballard BR Izban MG Jolin C Lammers PE Parrish DD Jr . A rapidly growing human papillomavirus-positive oral tongue squamous cell carcinoma in a 21-year old female: a case report. Oncol Lett. (2018) 15:7702–6. doi: 10.3892/ol.2018.8339, 29849799 PMC5962839

[11] Cheng WZ Wang WH Deng AP Dang X Liu C Wang X-C . Identification of an LDLR variant in a Chinese familial hypercholesterolemia and its relation to ROS/NLRP3-mediated pyroptosis in hepatic cells. J Geriatr Cardiol. (2023) 20:341–9. doi: 10.26599/1671-5411.2023.05.003, 37397863 PMC10308174

[12] Chen W Guo Z Li M Sheng W Huang G. Next-generation sequencing-based copy number variation analysis in Chinese patients with primary ciliary dyskinesia revealed novel DNAH5 copy number variations. Phenomics. (2024) 4:24–33. doi: 10.1007/s43657-023-00130-0, 38605905 PMC11003934

[13] Hiljadnikova-Bajro M Josifovski T Panovski M Dimovski AJ. A novel germline MLH1 mutation causing Lynch syndrome in patients from the Republic of Macedonia. Croat Med J. (2012) 53:496–501. doi: 10.3325/cmj.2012.53.496, 23100212 PMC3490460

[14] Heo Y Kim MH Kim DW Lee SA Bang S Kim MJ . Extent of pedigree required to screen for and diagnose hereditary nonpolyposis colorectal cancer: comparison of simplified and extended pedigrees. Dis Colon Rectum. (2020) 63:152–9. doi: 10.1097/dcr.0000000000001550, 31842160

[15] Ren C Liu Y Wang Y Tang Y Wei Y Liu C . Identification of novel Lynch syndrome mutations in Chinese patients with endometriod endometrial cancer. Cancer Biol Med. (2020) 17:458–67. doi: 10.20892/j.issn.2095-3941.2019.0295, 32587781 PMC7309470

[16] Hampel H Pearlman R Beightol M Zhao W Jones D Frankel WL . Assessment of tumor sequencing as a replacement for Lynch syndrome screening and current molecular tests for patients with colorectal cancer. JAMA Oncol. (2018) 4:806–13. doi: 10.1001/jamaoncol.2018.0104, 29596542 PMC5885168

[17] Pearlman R Frankel WL Swanson B Zhao W Yilmaz A Miller K . Prevalence and Spectrum of germline cancer susceptibility gene mutations among patients with early-onset colorectal cancer. JAMA Oncol. (2017) 3:464–71. doi: 10.1001/jamaoncol.2016.5194, 27978560 PMC5564179

[18] Peltomaki P Vasen H. Mutations associated with HNPCC predisposition—update of ICG-HNPCC/INSiGHT mutation database. Dis Markers. (2004) 20:269–76. doi: 10.1155/2004/305058, 15528792 PMC3839397

[19] Peltomaki P. Update on Lynch syndrome genomics. Fam Cancer. (2016) 15:385–93. doi: 10.1007/s10689-016-9882-8, 26873718 PMC4901089

[20] Kryukov GV Pennacchio LA Sunyaev SR. Most rare missense alleles are deleterious in humans: implications for complex disease and association studies. Am J Hum Genet. (2007) 80:727–39. doi: 10.1086/513473, 17357078 PMC1852724

[21] van der Klift HM Mensenkamp AR Drost M Bik EC Vos YJ Gille HJ . Comprehensive mutation analysis of PMS2 in a large cohort of probands suspected of Lynch syndrome or constitutional mismatch repair deficiency syndrome. Hum Mutat. (2016) 37:1162–79. doi: 10.1002/humu.23052, 27435373

[22] Suerink M van der Klift HM Ten Broeke SW Dekkers OM Bernstein I Capellá Munar G . The effect of genotypes and parent of origin on cancer risk and age of cancer development in PMS2 mutation carriers. Genet Med. (2016) 18:405–9. doi: 10.1038/gim.2015.83, 26110232

[23] González-Acosta M Del Valle J Navarro M Thompson BA Iglesias S Sanjuan X . Elucidating the clinical significance of two PMS2 missense variants coexisting in a family fulfilling hereditary cancer criteria. Fam Cancer. (2017) 16:501–7. doi: 10.1007/s10689-017-9981-1, 28365877

[24] Hu H Li H Jiao F Han T Zhuo M Cui J . Association of a novel point mutation in MSH2 gene with familial multiple primary cancers. J Hematol Oncol. (2017) 10:158. doi: 10.1186/s13045-017-0523-y, 28974240 PMC5627420

[25] Ribic CM Sargent DJ Moore MJ Thibodeau SN French AJ Goldberg RM . Tumor microsatellite-instability status as a predictor of benefit from fluorouracil-based adjuvant chemotherapy for colon cancer. N Engl J Med. (2003) 349:247–57. doi: 10.1056/NEJMoa022289, 12867608 PMC3584639

[26] Le DT Durham JN Smith KN Wang H Bartlett BR Aulakh LK . Mismatch repair deficiency predicts response of solid tumors to PD-1 blockade. Science. (2017) 357:409–13. doi: 10.1126/science.aan6733, 28596308 PMC5576142

[27] Evaristo G Harmath C Segal JP Shergill A Setia N. Diagnostic challenges due to a germline missense MSH2 variant in a patient with immunotherapy-responsive locally advanced rectal adenocarcinoma. Cancer Rep. (2024) 7:e70037. doi: 10.1002/cnr2.70037, 39696980 PMC11655914

[28] Ganesh K Stadler ZK Cercek A Mendelsohn RB Shia J Segal NH . Immunotherapy in colorectal cancer: rationale, challenges and potential. Nat Rev Gastroenterol Hepatol. (2019) 16:361–75. doi: 10.1038/s41575-019-0126-x, 30886395 PMC7295073

